# Role of *SaPCR2* in Zn Uptake in the Root Elongation Zone of the Zn/Cd Hyperaccumulator *Sedum alfredii*

**DOI:** 10.3390/life12050768

**Published:** 2022-05-23

**Authors:** Jun Ge, Jiayu Lin, Zhiying Wu, Kuan Xu, Jingyu Tao, Haizhong Lin, Shengke Tian, Lingli Lu

**Affiliations:** 1 Key Laboratory of Environment Remediation and Ecological Health, Ministry of Education, College of Environmental & Resource Science, Zhejiang University, Hangzhou 310058, China; gejun@zju.edu.cn (J.G.); 21714108@zju.edu.cn (J.L.); wuzhiying@zju.edu.cn (Z.W.); xukuan@zju.edu.cn (K.X.); taojingyu@zju.edu.cn (J.T.); tiansk@zju.edu.cn (S.T.); 2 Agricultural Technology Extension Center of Huangyan, Taizhou 318020, China; hyhaizi@163.com; 3 Zhejiang Provincial Key Laboratory of Subtropic Soil and Plant Nutrition, Zhejiang University, Hangzhou 310058, China

**Keywords:** gene function, ion transport and localization, overexpression, yeast function assays, plant morphology

## Abstract

Zn pollution is a potential toxicant for agriculture and the environment. *Sedum alfredii* is a Zn/Cd hyperaccumulator found in China and has been proven as a useful resource for the phytoremediation of Zn-contaminated sites. However, the molecular mechanism of Zn uptake in *S. alfredii* is limited. In this study, the function of SaPCR2 on Zn uptake in *S. alfredii* was identified by gene expression analysis, yeast function assays, Zn accumulation and root morphology analysis in transgenic lines to further elucidate the mechanisms of uptake and translocation of Zn in *S. alfredii*. The results showed that *SaPCR2* was highly expressed in the root elongation zone of the hyperaccumulating ecotype (HE) *S. alfredii*, and high Zn exposure downregulated the expression of *SaPCR2* in the HE *S. alfredii* root. The heterologous expression of *SaPCR2* in yeast suggested that SaPCR2 was responsible for Zn influx. The overexpression of *SaPCR2* in the non-hyperaccumulating ecotype (NHE) *S. alfredii* significantly increased the root uptake of Zn, but did not influence Mn, Cu or Fe. SR-μ-XRF technology showed that more Zn was distributed in the vascular buddle tissues, as well as in the cortex and epidermis in the transgenic lines. Root morphology was also altered after *SaPCR2* overexpression, and a severe inhibition was observed. In the transgenic lines, the meristematic and elongation zones of the root were lower compared to the WT, and Zn accumulation in meristem cells was also reduced. These results indicate that SaPCR2 is responsible for Zn uptake, and mainly functions in the root elongation zone. This research on *SaPCR2* could provide a theoretical basis for the use of genetic engineering technology in the modification of crops for their safe production and biological enhancement.

## 1. Introduction

Heavy metal pollution is a great agricultural and environmental concern in China [1,2,3]. Although zinc (Zn) is an essential component of the enzymes that are involved in protein synthesis, energy production, and in the maintenance of the structural integrity of biomembranes for both plants and humans [4,5,6], it could be potentially toxic at high concentrations and inhibit plant growth [7], as well as impact human health [8]. A nationwide survey in China showed that about 0.9% of the sampled soil was contaminated by Zn, based on the Chinese Soil Environmental Quality Standards [3]. Therefore, measures must be taken to control Zn pollution.

Hyperaccumulators can grow normally in metal-polluted sites and can accumulate large amounts of heavy metals in their shoots [9,10,11]. Owing to this unique and powerful ability, the phytoremediation of heavy-metal-polluted sites by hyperaccumulators has attracted considerable attention [10,12,13,14]. Previous studies have shown that the maximum uptake rate (vmax) in the hyperaccumulating ecotype (HE) *Sedum alfredii* was two-fold higher than that in the non-hyperaccumulating ecotype (NHE) [15,16]. This suggests that the high capacity of root uptake is a key process for metal hyperaccumulation [16].

It is well known that metal transporters that are localized in the root plasma membrane play an important role in metal uptake [17,18]. The role of many Zn transporters in plants has been identified, such as that of the plant cadmium resistance (PCR) family. This protein was first identified in *Arabidopsis thaliana* [19]. Studies have shown that both AtPCR1 and AtPCR2 proteins are localized in the plasma membrane [19,20]. The overexpression of *AtPCR1* in *A. thaliana* could increase Cd tolerance [19]. Additionally, AtPCR2 plays a role as a Zn efflux transporter and is mostly expressed in the epidermal cells and xylem of young roots, as well as in the epidermal cells of fully developed roots [20]. Interestingly, this family also participates in the regulation of organ size and cell numbers in fruit trees and crops [21,22].

For *S. alfredii*, complementary DNA-amplified fragment length polymorphism (cDNA-AFLP) analysis and transcriptome sequencing have been carried out [23,24], and several genes involved in Zn uptake have been analyzed. Yang et al. [25] indicated that SaZIP4, a Zrt/Irt-like protein (ZIP), is an important Zn uptake transporter in the roots and shoots of *S. alfredii*. In addition, SaNRAMP1 (natural resistance-associated macrophage protein) has been reported as a transporter in the NRAMP family that contributes to Zn accumulation in *S. alfredii* [26]. Moreover, SaMTP1, a metal tolerance protein, has been localized in the tonoplast and is responsible for Zn accumulation and tolerance [27]. In addition, the role of SaPCR2 in *S. alfredii* has also been investigated. It is localized in the plasma membrane and facilitates Cd efflux in the roots of transgenic plants [28]. However, the understanding of SaPCR2 in Zn uptake is still limited.

Therefore, in this study, we investigated the role of SaPCR2 in Zn uptake in *S. alfredii*, mainly by analyzing gene expression, yeast function assays, Zn accumulation and root morphology in transgenic lines. Through this research, we hope to provide a new theoretical basis for Zn uptake in *S. alfredii*.

## 2. Materials and Methods

### 2.1. Plant Material and Culture Conditions

Seedlings of the HE and NHE *S. alfredii* were collected from an abandoned Pb/Zn mine in Quzhou and from a tea plantation in Hangzhou (both in Zhejiang Province, China), respectively. HE plants can tolerate and hyperaccumulate 9000 mg kg^−1^ Cd and 19,670 mg/kg^−1^ Zn in the shoot, while NHE plants cannot tolerate heavy metals [23,29,30]. Healthy and uniform branches of HE and NHE *S. alfredii* were pre-cultivated in deionized water for root growth. After two weeks, the seedlings were subjected to a 4-day exposure in one-fourth-strength and one-half-strength nutrient solution, and then cultured in full-strength nutrient solution. The full-strength nutrient solution contained 2.0 mM Ca^2+^, 4.0 mM NO_3_^−^, 1.6 mM K^+^, 0.1 mM H_2_PO_4_^−^, 0.5 mM Mg^2+^, 1.2 mM SO_4_^2−^, 0.1 mM Cl^−^, 10 μM H_3_BO_3_, 0.5 μM MnSO_4_, 5.0 μM ZnSO_4_, 0.2 μM CuSO_4_, 0.01 μM (NH_4_)_6_Mo_7_O_24_ and 20 μM EDTA-NaFe. The nutrient solution was continuously aerated and renewed every 3 d. Nutrient solution pH was measured using a pH meter (Sartorius PB-10, Germany) and adjusted to 5.5–5.8 daily using 0.1 N HCl. Plants were grown in a growth chamber with a 16/8 h photoperiod at 400 μmol m^−2^ s^−1^, a day/night temperature of 26 °C/20 °C, and a day/night humidity of 70%/85%.

### 2.2. Expression Pattern of SaPCR2 in S. alfredii

After pre-culturing in nutrient solution for 3 weeks, the roots (including 0 to 1 cm and 1 to 2 cm from the root tip) and shoots of HE *S. alfredii* with 3 replicas were separated. In a separate experiment, another batch of HE and NHE *S. alfredii* with 3 replicas were exposed to basal nutrient solution with 200 μM ZnSO4 addition or Zn deficiency for 7 d, and the roots were separated. Then, 25 to 50 mg of each part of the plant samples were dried with a paper towel, immediately frozen in liquid nitrogen, and ground into powder. Then, the total RNA of each sample was extracted using a Spin Column Plant Total RNA Purification Kit (Sangon Biotech, Shanghai, China), and converted to single-strand cDNA using HiScript II Q RT SuperMix for qPCR (+gDNA wiper) (Vazyme, Nanjing, China). Gene expression patterns were determined using quantitative real-time PCR with ChamQTM SYBR Color qPCR Master Mix (without Rox) (Vazyme, Nanjing, China). The constitutively expressed *SaActin1* gene was used as an internal standard [24]. The primers designed for the real-time PCR analysis are listed in Table S1. Relative gene expression was calculated using the 2^−∆∆CT^ method. Sequence data of *SaPCR2* can be found in the GenBank under the accession number of HE728063.1 [28].

### 2.3. Yeast Expression Analysis

**Construction of yeast transgenic:** The pDR196-SaPCR2 vector was constructed as previously described [28]. Primers used for the plasmid construction are listed in Table S1. To analyze the effect of SaPCR2 on Zn uptake and tolerance, both pDR196-SaPCR2 and pDR196 empty vectors were transformed into yeast (*Saccharomyces cerevisiae*) strains, including the Zn/Cd-sensitive mutant Δ*zrc1* (*MATα*; *his3*; *leu2*; *met15*; *ura3*; *YMR243c::kanMX4*) and the Zn-absorption-deficient mutant ZHY3 (*MATα*; *zrt1::LEU2*; *zrt2::HIS3*; *ade6*; *can1*; *his3*; *leu2*; *trp1*; *ura3*) by the LiAc/PEG/ssDNA method [31]. The positive strains were screened out by selected solid synthetic dropout (SD) media (absence of uracil) and verified by PCR.

**Growth analysis of yeast transgenic:** For the Zn tolerance assay, both Δ*zrc1* and ZHY3 yeast cells were cultured in selected liquid SD media until the OD_600_ reached 1.0. Then, 5 μL serial dilutions (OD600 = 1.0, 0.1, 0.01, and 0.001) were spotted on solid SD media with different Zn concentrations (for Δ*zrc1*: CK (0 mM), 0.5 mM, 1.0 mM, 2.0 mM, and 4.0 mM ZnSO_4_; for ZHY3: CK (200 μM ZnSO_4_) and -Zn (Zn deficiency, 200 μM ZnSO_4_ + 200 μM EDTA)) at 30 °C. Photographs were taken after a 3-day incubation period. Drop tests were repeated three times with similar results. To investigate the growth curve of Δ*zrc1* yeast cells under Zn stress, the OD_600_ value with 3 replicas was recorded after 6 h, 12 h, 24 h, 48 h and 72 h with Zn treatments (CK (0 mM) and 2.0 mM ZnSO_4_) in liquid SD media using a photometer (Eppendorf AG22331, Hamburg, Germany).

**Zn accumulation analysis in yeast transgenic:** In addition, for Zn accumulation analysis, Δ*zrc1* with 3 replicas was cultured overnight, diluted to OD_600_ = 0.1 in the liquid SD media with different Zn concentrations (2.0 mM and 4.0 mM ZnSO_4_) for 24 h at 30 °C with an oscillation of 200 rpm. The yeast cells were collected via centrifugation and washed three times with 10 mM EDTA-2Na. The cells were dried for 2 d at 65 °C and digested in HNO_3_-H_2_O_2_ to determine the Zn concentration using an atom absorption spectrum (SP-5320AA, Spectrum Instruments, Shanghai, China).

### 2.4. Heavy Metal Concentration Analysis in the SaPCR2 Overexpression of NHE S. alfredii

The *SaPCR2* overexpression lines of NHE *S. alfredii* (L1 and L2) were obtained in our previous study by *Agrobacterium tumefaciens* transformation [28]. After pre-culturing in nutrient solution for 3 weeks, both wild type (WT) NHE *S. alfredii* and *SaPCR2* overexpression lines (L1 and L2) with 3 replicas were treated with 50 μM ZnSO_4_ for 14 d. In a separate experiment, five-week-precultured WT, L1, and L2 lines in basal nutrient solution with 3 replicas were used to determine the uptake of trace elements including Zn, Mn, Cu and Fe. After harvest, plant samples were rinsed with tap water, soaked in 5 mM EDTA-2Na for 15 min to remove surface-absorbed ions, rinsed with deionized water, and dried using a paper towel. Roots and shoots were separated and oven-dried at 65 °C for 72 h. Elemental concentrations were analyzed using ICP-MS (Perkin Elmer Sciex Elan DRC-e, Waltham, MA, USA) after digestion with 5 mL of HNO_3_ and 1 mL of H_2_O_2_ at 180 °C for 8 h. Each treatment had three replicates with individual plants.

### 2.5. SR-μ-XRF Analysis Revealed the Zinc Distribution in the SaPCR2-Overexpressing NHE S. alfredii

After pre-culturing in nutrient solution for 3 weeks, both WT NHE *S. alfredii* and *SaPCR2* overexpression lines (L1 and L2) were treated with 10 μM and 50 μM ZnSO_4_ for 14 d, respectively. The plant samples were rinsed with tap water, soaked in 5 mM EDTA-2Na for 15 min to remove surface-absorbed ions, rinsed with deionized water, and dried using a paper towel. The root samples were separated, frozen in liquid nitrogen immediately, and freeze-dried at −20 °C (LGJ-15E, Foring Technology Development, Beijing, China). Intact roots were selected and pasted on sulfur-free tape. In addition, the cross-sections of the stems (60 μm) were sliced (Thermo Cryostar NX50, Thermo Scientific, Waltham, MA, USA) and also freeze-dried at −20 °C (LGJ-15E, China). The samples were then subjected to synchrotron radiation-based micro-X-ray fluorescence (SR-μ-XRF) analysis at the Stanford Synchrotron Radiation Lightsource (SSRL, Menlo Park, CA, USA). The SR-μ-XRF technique was employed using a Si (220) double-crystal monochromator with a storage ring SPEAR-3 containing 90–100 mA at 3.0 GeV. The distribution patterns of Zn were collected at the SSRL beamline 2–3 using an X-ray beam of 5 µm pixel size in 10 μm steps and 200 ms dwell time for the intact root tip samples, and in 5 μm steps and 200 ms dwell time for the stem cross-section samples.

### 2.6. Root Morphology in the SaPCR2-Overexpressing NHE S. alfredii

To determine the root elongation under Zn stress, the newly rooted WT, L1, and L2 plants with 8 replicas were transplanted to a nutrient solution with 50 μM ZnSO_4_, and the root length was measured at 1, 2, 4, 6, 8, 10, 12, and 14 d. In addition, to analyze the effect of Zn treatment on the root morphology of *SaPCR2*-overexpressing lines, WT, L1, and L2 plants with 8 replicas were cultured in basal nutrient solution with 5 μM (CK), 10 μM, and 50 μM ZnSO_4_ for 14 d. The root phenotype was recorded using a root scanner (Epson Perfection V850 Pro, Epson, Nagano, Japan). The total root length, total root surface area and total root volume were calculated using the WinRHIZO 2017 Pro software (Regent Instruments, Québec, QC, Canada). The lateral root density was calculated using Equation (1):

(1) $$Lateral\;root\;density\;\left(\mathrm{number}\cdot {\mathrm{cm}}^{-1}\right)=\frac{number\;of\;lateral\;root}{taproot\;length},$$

in which the taproot length was measured using ImageJ (Version 1.53e, NIH image, Bethesda, ML, USA). The relative total root elongation length was calculated using Equation (2):

(2) $$Relative\;total\;root\;elongation\;length\;\left(\%\right)=\frac{root\;elongation\;length\;at\;treatment\;condition}{root\;elongation\;length\;at\;control\;condition}\times 100\%,$$

The relative total root surface and relative total root volume were calculated using methods similar to that of the relative total root elongation length. In addition, the WT, L1, and L2 plants with 5 replicas were cultured in a basal nutrient solution with 10 μM ZnSO_4_ for 14 d, and the root tip structures were photographed with a microscope (Nikon ECLIPSE Ni-E, Tokyo, Japan) using a NEO-5.5-CL3 (Andor, EU) camera at 20x magnification, and the root length of the elongation zone and meristematic zone were measured using ImageJ (Version 1.53e, NIH image, Bethesda, ML, USA).

### 2.7. Statistical Analysis

All data were statistically analyzed using a data processing system (DPS, Taizhou, China). Analysis of variance and mean separation were performed using the *t*-test or ANOVA with the least significant difference (LSD) method at *p* < 0.05 and *p* < 0.01. The visualization of data from the micro-X-ray fluorescence was performed using the SMAK software (Version 1.4, https://www.sams-xrays.com/smak, accessed on 10 August 2020) [32].

## 3. Results

### 3.1. Expression Pattern of SaPCR2 in the HE and NHE S. alfredii

**Figure 1:**
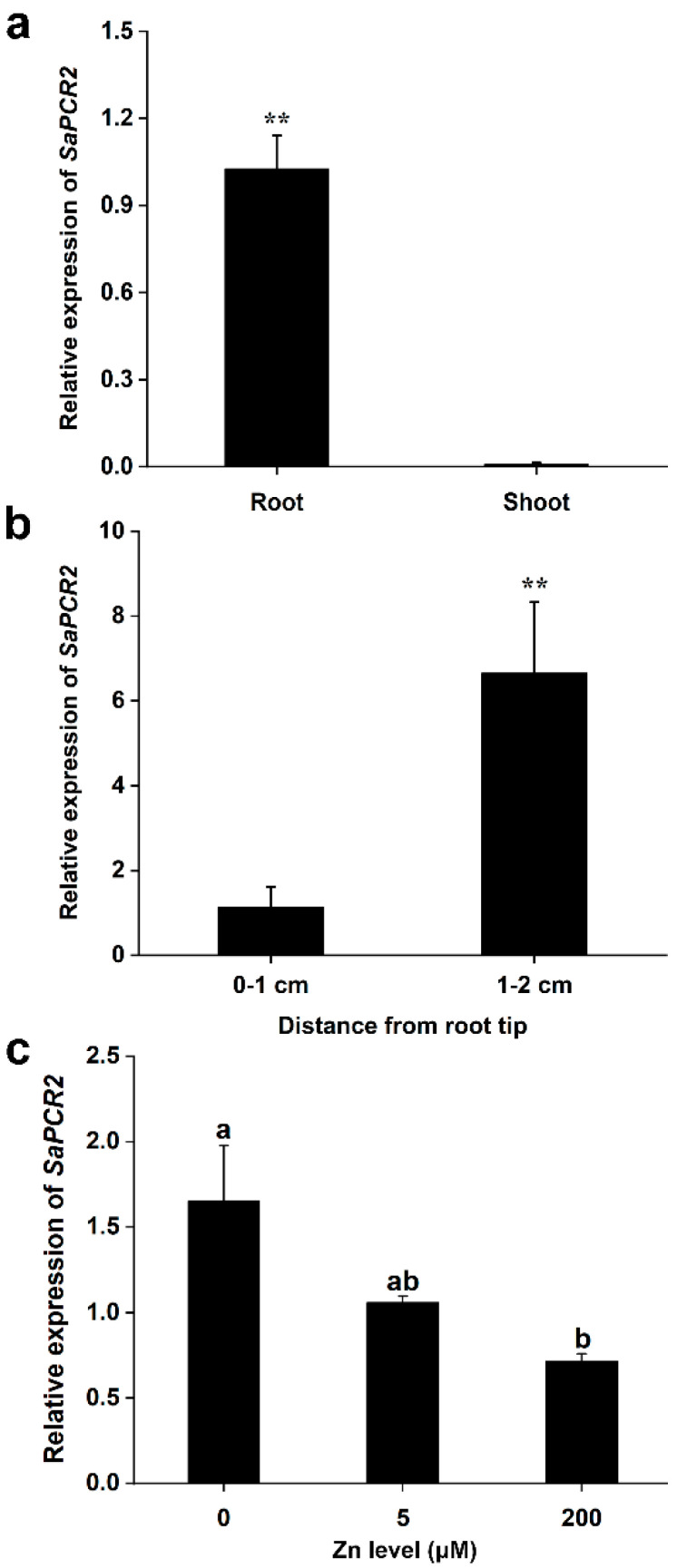
Expression pattern of *SaPCR2* in the hyperaccumulating ecotype (HE) *Sedum alfredii*. (**a**) Expression levels of *SaPCR2* in the root and shoot of the HE *S. alfredii* after a three-week-preculture. (**b**) Expression levels of *SaPCR2* at the root (0 to 1 cm and 1 to 2 cm), from the root tip of the HE *S. alfredii* after a three-week-preculture. (**c**) Expression levels of *SaPCR2* in the root of the HE *S. alfredii* without Zn (0 μM) or with an additional 200 μM ZnSO_4_ for 7 d. ** indicates significant difference between various organs or tissues (*p* < 0.01) by *t*-Test. Different letters indicate significant differences between treatments (*p* < 0.05) by LSD. Error bars = mean ± standard error (SE; *n* = 3).

Real-time PCR was used to analyze the expression pattern of *SaPCR2* in the HE and NHE *S. alfredii*. After pre-culturing in nutrient solution for 4 weeks, *SaPCR2* was more highly expressed in the roots of HE *S. alfredii* than in the shoots (*p* < 0.01, Figure 1a). In addition, the expression level of *SaPCR2* at 1 to 2 cm from the root tip was approximately six times higher than that of 0 to 1 cm (*p* < 0.01, Figure 1b). Furthermore, Zn treatment was applied to explore the regulation of *SaPCR2* expression in the roots of HE and NHE *S. alfredii*. It was found that the expression level of *SaPCR2* in HE *S. alfredii* was downregulated with increasing exogenous Zn concentrations (Figure 1c). However, under Zn deficiency conditions, the expression of *SaPCR2* in HE *S. alfredii* was upregulated 1.6 times compared to that under 200 μM Zn exposure (*p* < 0.05, Figure 1c). Additionally, in our previous study, we analyzed the expression level of *SaPCR2* in NHE *S. alfredii*, and the expression was extremely low compared with HE, which were both grown in normal nutrient solution [28]. In this current study, we also investigated how *SaPCR2* responded to high Zn treatment and Zn deficiency in NHE *S. alfredii*. However, the expression of *SaPCR2* in NHE was too low to be detected (data not shown).

### 3.2. Functional Analysis of SaPCR2 in Yeast

**Figure 2:**
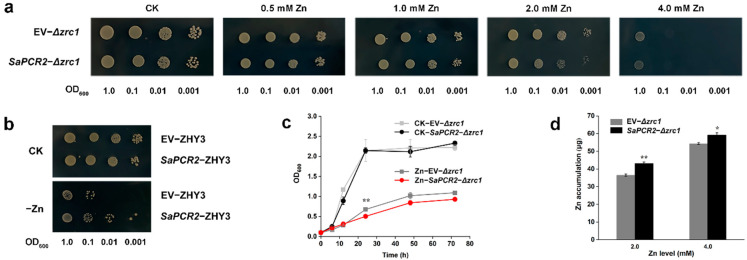
Functional analysis of the heterologous expression of SaPCR2 in the *Saccharomyces cerevisiae* mutant strain. (**a**) Growth of Zn/Cd-sensitive yeast strain Δ*zrc1* transformed with pDR196 empty vector (labeled as EV-Δ*zrc1*) or SaPCR2 vector (labeled as *SaPCR2-*Δ*zrc1*). (**b**) Growth of Zn-absorption-deficient yeast strain ZHY3, transformed with pDR196 empty vector (labeled as EV-*ZHY3*) or SaPCR2 vector (labeled as *SaPCR2-ZHY3*), diluted to OD600 = 1.0, 0.1, 0.01, and 0.001, then dropped on solid synthetic dropout (SD) medium with different Zn levels. Plates were incubated for 3 d at 30 °C. Drop tests were repeated three times with similar results. (**c**) Growth curves of EV-Δ*zrc1* and *SaPCR2-*Δ*zrc1* in SD liquid medium with or without 2 mM ZnSO_4_. (**d**) Zn accumulation of EV-Δ*zrc1* and *SaPCR2-*Δ*zrc1* in the liquid SD medium with 2.0 mM or 4.0 mM ZnSO_4_ for 24 h at 30 °C. * and ** indicate significant difference to the empty vector transformants under the same treatment at *p* < 0.05 by LSD and *p* < 0.01 level by *t*-test, respectively. Error bars = mean ± standard error (SE; *n* = 3).

As shown in Figure 2a, when the Zn concentrations in the solid SD media (absence of uracil) were less than 1 mM, both samples of Δ*zrc1* transformed with the pDR196 empty vector (labeled as EV-Δ*zrc1*) and the pDR196-SaPCR2 vector (labeled as *SaPCR2*-Δ*zrc1*) were healthy and similar. When the Zn concentration was increased to 2 mM, the growth of Δ*zrc1* transferred with the pDR196-SaPCR2 vector (labeled as *SaPCR2*-Δ*zrc1*) was visibly inhibited, compared with those transferred with the pDR196 empty vector (labeled as EV-Δ*zrc1*) (Figure 2a). On the other hand, the ability to grow under zinc-deficient conditions was restored in the ZHY3 mutant, which was transferred with SaPCR2 (Figure 2b).

To further verify the influence of SaPCR2 on the Zn tolerance of the Δ*zrc1* mutant, liquid SD media (absence of uracil) culture was used to explore the growth of yeast. The results showed that, after treatment with 2 mM ZnSO_4_ for 24 h, the OD_600_ value of Δ*zrc1* transferred with the pDR196-SaPCR2 vector (labeled as *Zn*-*SaPCR2*-Δ*zrc1*) was significantly (*p* < 0.01) lower than those transferred with the pDR196 empty vector (labeled as Zn-EV-Δzrc1, Figure 2c). Furthermore, the results of Zn content determination showed that the heterologous expression of SaPCR2 in Δ*zrc1* significantly (*p* < 0.05) increased Zn content by 18% and 7% after treatment with 2 mM ZnSO_4_ and 4 mM ZnSO_4_, respectively (Figure 2d).

### 3.3. Effect of SaPCR2 Overexpression on Zn Concentration in the NHE S. alfredii

**Figure 3:**
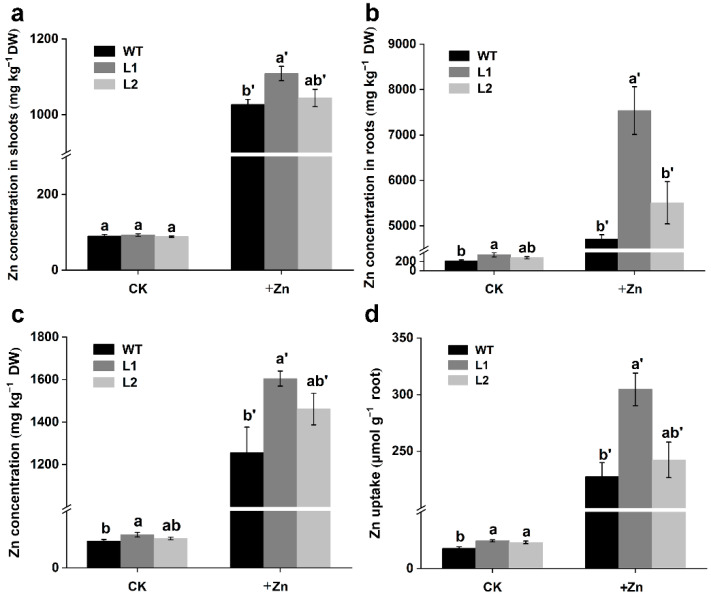
(**a**–**c**) Zn concentration and (**d**) Zn uptake in the non-hyperaccumulating ecotype (NHE) *Sedum alfredii* wild type (WT) and *SaPCR2*-overexpressing lines (L1 and L2). Three-week-precultured plants were transferred to a basal nutrient solution with or without additional 50 μM ZnSO_4_. Zn concentrations in the (**a**) shoots, (**b**) roots, and (**c**) whole plants were determined after a 14-day treatment. Total Zn uptake was calculated in terms of the root dry weight. Different letters indicate significant differences between genotypes in a treatment (*p* < 0.05). Error bars = mean ± standard error (SE; n = 3).

To reveal the effect of *SaPCR2* overexpression on the Zn content in plants, three-week-old WT and *SaPCR2*-overexpressed (L1 and L2) NHE *S. alfredii* seedlings were exposed to a treatment of 50 μM ZnSO_4_ in the nutrient solution for 14 d. It was found that the Zn content in the plants increased with the exogenous Zn addition (Figure 3). Under the 50 μM ZnSO_4_ treatment, the Zn content in the shoots and roots of the *SaPCR2* overexpression lines was higher than that of the WT (*p* < 0.05, Figure 3a,b). Even under control conditions (5 μM ZnSO_4_), the overexpression lines had higher Zn content in the roots (*p* < 0.05, Figure 3b). In addition, the Zn concentration in the whole plant was 16 to 28% higher in the transgenic lines than in the WT under 50 μM ZnSO_4_ treatment (*p* < 0.05, Figure 3c). The Zn uptake in the roots of the transgenic lines was also higher than that in the WT (*p* < 0.05, Figure 3d). In a separate experiment, the trace element uptake in the five-week-precultured WT, L1, and L2 lines was measured, and it was only Zn that significantly (*p* < 0.05) increased in the transgenic lines, rather than Mn, Cu or Fe (Figure S1).

### 3.4. Effect of SaPCR2 Overexpression on Zn Distribution in the NHE S. alfredii

**Figure 4:**
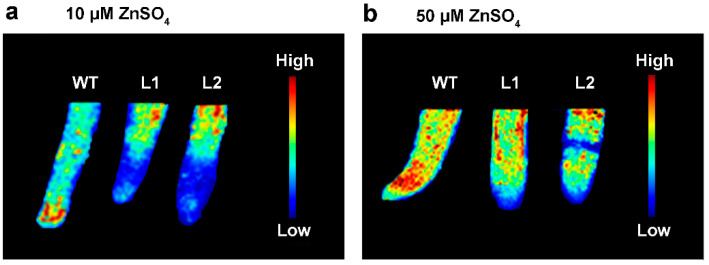
Synchrotron radiation-based micro-X-ray fluorescence (SR-μ-XRF) mapping of Zn in the root tips of the non-hyperaccumulating ecotype (NHE) *Sedum alfredii* wild type (WT), and *SaPCR2*-overexpressing lines (L1 and L2) after exposure to (**a**) 10 μM ZnSO_4_ or (**b**) 50 μM ZnSO_4_ for 14 d. Pixel brightness is displayed in RGB, with the brightest spots corresponding to the highest element fluorescence.

Synchrotron radiation-based micro-X-ray fluorescence (SR-μ-XRF) technology was used to investigate the effect of *SaPCR2* overexpression on Zn distribution in the NHE *S. alfredii* intact root tips and stem cross-sections. Under treatments of 10 μM and 50 μM ZnSO_4_ for 14 d, the XRF images showed that Zn was mainly distributed in the root meristematic zone of the WT (Figure 4). In contrast, Zn accumulated much more in the elongation region of the *SaPCR2* overexpression lines than in the WT (Figure 4).

**Figure 5:**
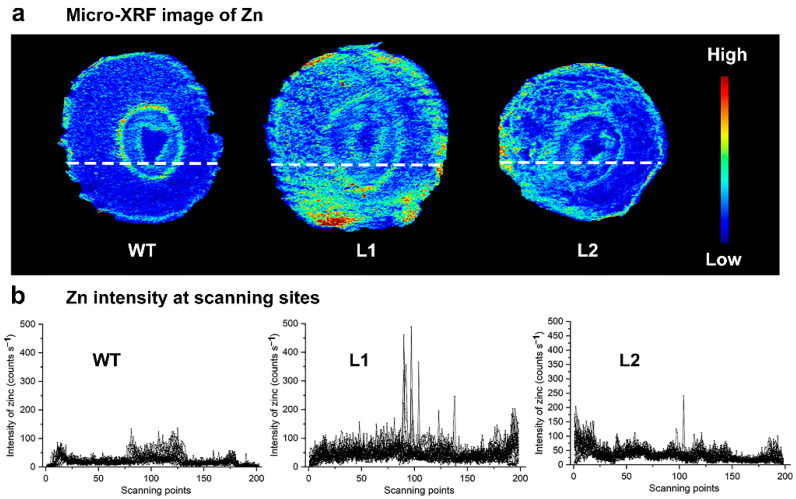
(**a**) Synchrotron radiation-based micro-X-ray fluorescence (SR-μ-XRF) image of Zn in the stem cross-sections (60 μm) of the non-hyperaccumulating ecotype (NHE) *Sedum alfredii* wild type (WT) and *SaPCR2*-overexpressing lines (L1 and L2) after exposure to 50 μM ZnSO_4_ for 14 d. Pixel brightness is displayed in RGB, with the brightest spots corresponding to the highest element fluorescence. (**b**) Zn intensity (counts s^−1^) at the scanning sites is marked as dashed lines in (**a**).

In the stem of the WT, the Zn signal was weak and restricted to the vascular buddle tissues (Figure 5a). However, in the stems of the transgenic lines, a strong Zn signal was detected and widely distributed in the vascular buddle tissues, cortex and epidermis (Figure 5a). As shown in Figure 5b, Zn intensity counts in the scanning sites of L1 and L2 were apparently higher than those of the WT, especially in the vascular buddle tissues.

### 3.5. Root Morphology in the SaPCR2-Overexpressing NHE S. alfredii

**Figure 6:**
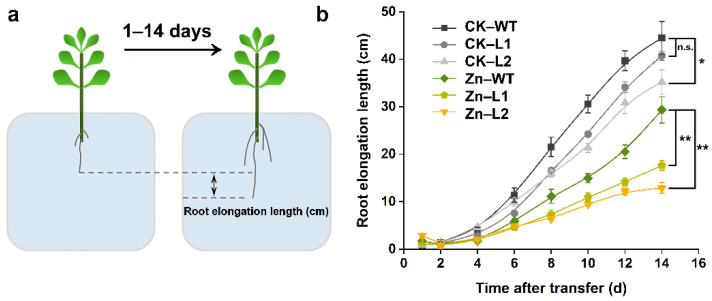
Effects of Zn on the root elongation of the non-hyperaccumulating ecotype (NHE) *Sedum alfredii* wild type (WT) and *SaPCR2*-overexpressing lines (L1 and L2). The newly rooted plants were transferred to a basal nutrient solution with or without additional 50 μM ZnSO_4_. The root elongation length was measured at 1, 2, 4, 6, 8, 10, 12 and 14 d. (**a**) Diagram of the root elongation measurement method. (**b**) Time-course of the root elongation length under Zn exposure. * and ** indicate significant differences between genotypes at *p* < 0.05 and *p* < 0.01, respectively. ns—not significant. Error bars = mean ± standard error (SE; n = 8).

Newly rooted WT and *SaPCR2*-overexpressing NHE *S. alfredii* plants were transplanted into a nutrient solution with 50 μM ZnSO_4_. Root elongation length was measured at 1, 2, 4, 6, 8, 10, 12, and 14 d, following the method shown in Figure 6a. The results showed that, compared to the control condition, Zn addition inhibited the root elongation of both WT and transgenic lines, but it was much more severe in the latter (Figure 6b).

**Figure 7:**
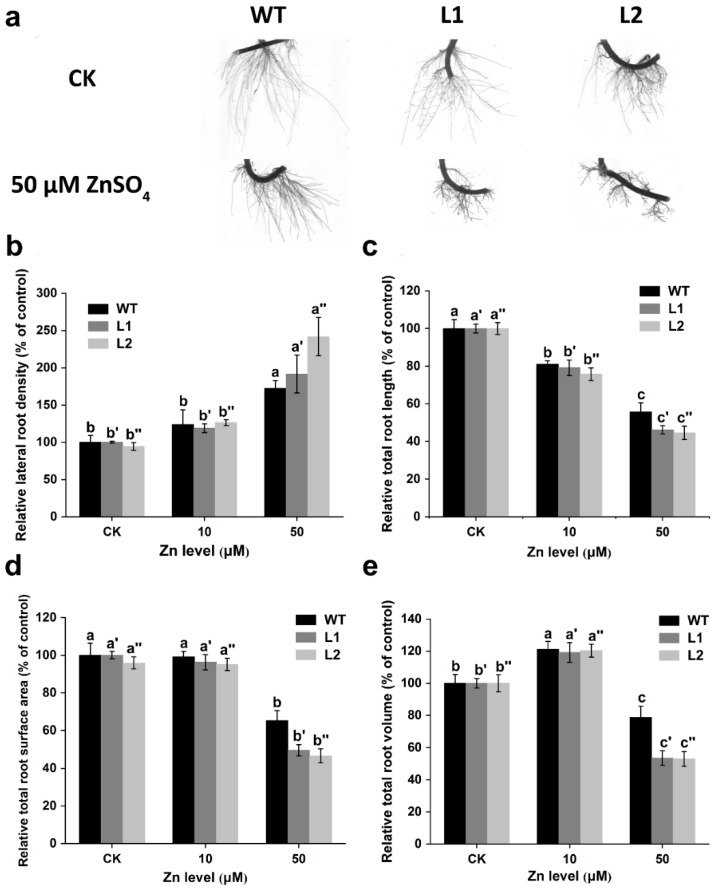
Effects of Zn on the root morphology of the non-hyperaccumulating ecotype (NHE) *Sedum alfredii* wild type (WT) and *SaPCR2*-overexpressing lines (L1 and L2). The plants were cultured in a basal nutrient solution with 5 μM (as CK), 10 μM, and 50 μM ZnSO_4_ for 14 d, respectively. Root morphologies were analyzed, including (**a**) the growth of roots, (**b**) relative total lateral root density, (**c**) relative total root elongation length, (**d**) relative total root surface area, and (**e**) relative total root volume. Different letters indicate significant differences between treatments in the same genotype. Error bars = mean ± standard error (SE; n = 8).

After treatment with 50 μM ZnSO_4_ for 14 d, the overall growth conditions were largely inhibited in the transgenic lines (L1 and L2), which were presented as thick and short roots (Figure 7a). Furthermore, selected root morphology indicators were measured in both the WT and transgenic lines, including relative lateral root density, relative total root length, relative total root surface area, and relative total root volume (Figure 7b–e). The results showed that, with the addition of exogenous ZnSO_4_, the inhibition of root growth became significant (*p* < 0.05, Figure 7b–e). Specifically, the relative total root length, relative total root surface area, and relative total root volume were all decreased by 46% to 55% in the transgenic lines under 50 μM ZnSO_4_, while they were only decreased by 21% to 44% in the WT (Figure 7b–e).

**Figure 8:**
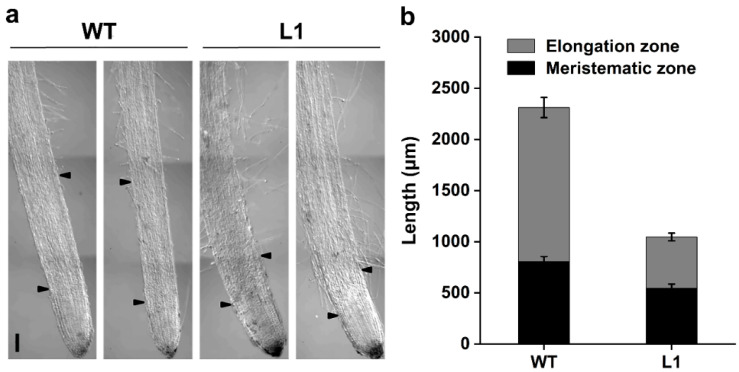
Root tip structures of the non-hyperaccumulating ecotype (NHE) *Sedum alfredii* wild type (WT) and *SaPCR2*-overexpressing lines (L1 and L2) under Zn treatment. The plants were exposed to 10 μM ZnSO_4_ for 14 d. (**a**) Growth of root tips. Bar = 200 μm. (**b**) Length of meristematic zone and elongation zone. Error bars = mean ± SE (*n* = 5).

Root tip structures were also observed with or without 10 μM ZnSO_4_ treatment for 14 d (Figure 8a and Figure S2a). The results indicated that the elongation zone length and meristematic zone length were both decreased in the transgenic lines compared with those of the WT, mainly attributed to *SaPCR2* overexpression, regardless of whether Zn was added (Figure 8b and Figure S2b). Specifically, the effect was much more obvious in the elongation zone (Figure 8b and Figure S2b).

## 4. Discussion

### 4.1. SaPCR2 Is Responsible for Zinc Uptake in S. alfredii

It is generally acknowledged that there are two transport systems that mediate Zn uptake in plants, including the high-affinity transport system under low Zn conditions and the low-affinity transport system under high Zn conditions [33]. For hyperaccumulators, the uptake of heavy metals by root cells is of great importance for metal hyperaccumulation [34]. In the present study, it was found that SaPCR2 also exhibited the ability to absorb Zn, which could be supported in three aspects, according to our experiments.

First, the expression level of *SaPCR2* in HE *S. alfredii* was upregulated under Zn deficiency conditions (Figure 1c), which indicated that Zn deficiency could stimulate the expression of *SaPCR2* in HE *S. alfredii* to enhance the absorption of external Zn. Meanwhile, high Zn treatment (200 μM) had little impact on the expression level of *SaPCR2*, which was possibly because the plants already accumulated high Zn [15,35]. Second, after SaPCR2 was transferred into the Zn/Cd-sensitive mutant Δ*zrc1*, the growth of yeast was inhibited under high Zn treatment and more Zn accumulated (Figure 2a,c,d), showing that SaPCR2 has Zn transport ability and enhanced sensitivity to Zn stress. Consistently, the expression of SaPCR2 in the Zn-uptake-deficient mutant ZHY3 restored strain growth (Figure 2b). This result is consistent with that of the high-affinity Zn transporter NcZNT1 in yeast mutants [36,37]. In our previous study, we found that SaPCR2 was localized in the plasma membrane [28]. Therefore, we presume that SaPCR2 is a Zn transporter responsible for Zn influx in cells. Third, the overexpression of *SaPCR2* increased the Zn content in NHE *S. alfredii* under both control (5 μM) and high Zn (50 μM) treatment conditions (*p* < 0.05, Figure 3). Therefore, according to the three strands of evidence, we proposed that SaPCR2 is responsible for Zn uptake in *S. alfredii* roots.

### 4.2. SaPCR2 Mediates Zinc Transport in the Root Elongation Zone of S. alfredii

It has been widely reported that most genes in the PCR family are highly expressed in the roots, but the localization of some gene members could be different in diverse parts of the same plant species. For instance, in the elongation zone of Arabidopsis thaliana, AtPCR2 is localized in the vascular buddle tissues and epidermis, whereas in the maturation zone, this protein is mainly localized in the epidermis [20]. This allows AtPCR2 to have two independent functions in Arabidopsis roots: promoting Zn loading in the xylem, and reducing Zn accumulation in the epidermal cells [20]. Besides, highly homogenous genes may have distinct expression patterns in various plant species. For example, NRAMP5 is responsible for the transverse transport of Mn and Cd in both *O. sativa* and *Hordeum vulgare*, but OsNRAMP5 is localized in the endodermis and epidermis of basal roots [38] and NvNRAMP5 is localized in the epidermis and in the cell membrane of root tips [31].

To further reveal the role of SaPCR2 in Zn transport in plant roots, series hydroponic culture experiments were performed in the *SaPCR2*-overexpressing NHE *S. alfredii* plants. The results showed that, compared to the WT, high Zn treatment led to a more severe inhibition effect on the transgenic lines (Figure 6 and Figure 7). The observation of root structures by microscopy showed that the overexpression of *SaPCR2* inhibited the growth of the root elongation zone and part of the region in the maturation zone (Figure 8). Generally, metabolism in the root meristematic zone is active, and the cells in this region have a strong absorption ability for nutrients. However, vascular buddle tissues have not yet been formed, and the transverse transport of nutrients in the root meristematic zone is limited. Because of the Casparian strip in the region near the root hairs, nutrients enter the stele through the symplasm where the parenchymal cells next to the vascular buddle tissues are of great importance. In this study, we found that the expression level of *SaPCR2* was higher at a greater distance from the root tips (Figure 1b). In addition, the distribution of Zn in the root tip meristematic zone in the transgenic lines was much less than that that of the WT (Figure 4). Thus, we speculate that the overexpression of *SaPCR2* probably increased the Zn uptake in the parenchyma cells in plants, thereby enhancing the transverse transport of Zn in the roots and decreasing the accumulation of Zn in the meristematic zone.

### 4.3. The Overexpression of SaPCR2 Increased Zinc Concentration in the Shoots of S. alfredii

Many Zn transporters are not only involved in the transverse transport of Zn at the root, but also participate in its root-to-shoot translocation. For instance, NcZNT1 (a member of the ZIP family) is localized in the epidermis and was largely distributed in the vascular buddle tissues, which suggests that this protein may be related to the long-distance transport of heavy metals [36]. Similarly, in our study, we found that the Zn concentration in the *SaPCR2* overexpression lines was much higher than that of the WT (*p* < 0.05, Figure 3). The Zn distribution pattern in stems revealed by the SR-μ-XRF analysis verified this result (Figure 5). In the WT plants, Zn was mainly restricted in the vascular buddle tissues while, in the transgenic lines, Zn was distributed in the vascular buddle tissues as well as in the cortex and epidermis (Figure 5). It is likely that the overexpression of *SaPCR2* increases the Zn uptake in the roots and translocates more Zn into the shoots.

Such understanding of the function of SaPCR2 could be used in two aspects. First, we could overexpress *SaPCR2* in crops, which may also increase Zn accumulation for bio-enhancement while decreasing Cd accumulation, as in the NHE *S. alfredii*. Second, in the present study, we only overexpressed *SaPCR2* in NHE *S. alfredii*; in future experiments, we would like to overexpress *SaPCR2* in HE *S. alfredii*. With the great ability of root-to-shoot translocation and metal detaxation in HE *S. alfredii*, the overexpression of *SaPCR2* may enhance phytoremediation efficiency in Zn-polluted sites.

### 4.4. SaPCR2 Participates in Zn and Cd Transport, but May Not Participate in Mn, Cu or Fe Transport

Many Zn transporters have also been reported to participate in the transport of other metals such as those of Fe, Mn, Cu and Cd. For example, AtZIP1 and AtZIP2 can transport both Zn and Mn, and the knockout of these genes by T-DNA insertion has reduced the translocation of Zn and Mn to the shoots [39]. In addition, AtZIP2 localizes in the stele and plasma membrane of the roots and acts on the transport of Zn and Mn to the stele and xylem parenchyma cells [39]. In *O. sativa*, OsPCR1 is responsible for the transport and distribution of heavy metals [40]. The knockout of *OsPCR1* could increase the Zn content in grains and reduce the Mn and Cd content in rice [40].

In our previous study, we found that the overexpression of *SaPCR2* reduced the Cd content in NHE *S. alfredii* [28]. In the present study, we further investigated other element contents in the overexpression lines grown in basal nutrient solution. The results showed that the overexpression of *SaPCR2* reduced the root uptake of Zn, while concentrations of Mn, Cu and Fe contents did not differ significantly between the transgenic lines and the WT (Figure S1). This indicates that SaPCR2 is a Zn and Cd transporter, but may not be a transporter of Mn, Cu or Fe. Additionally, this needs further verification under Mn, Cu and Fe treatments. We also compared the protein sequences between SaPCR2, AtZIP1, AtZIP2 and NcZNT1 (Figure S3), as they all showed Zn transport ability. This showed that SaPCR2 had low identity with the other 3 proteins (from 7.12% to 9.07%). After aligning these four protein sequences together, we found a probably conserved domain “GXXXTGLXH”, as marked with a red box in Figure S3. This highly conserved sequence region may be responsible for Zn transport, but this needs further confirmation. As far as we know, this is the first identified transporter the participates in both Cd exclusion and Zn uptake. We assume that SaPCR2 may work like the Na^+^/H^+^ antiporter, Ca^2+^/H^+^ antiporter and Ca^2+^/Na^+^ antiporter [41,42,43]. To be specific, SaPCR2 may uptake a Zn^2+^ ion while exporting a Ca^2+^ ion. Additionally, as Ca^2+^ and Cd^2+^ showed similar function in *S. alfredii* [44,45,46,47], SaPCR2 thus shows Cd export ability while also showing Zn uptake ability in NHE *S. alfredii*. However, this viewpoint needs to be verified by further experimental evidence.

## 5. Conclusions

*SaPCR2* was highly expressed in the root elongation zone of *S. alfredii*, and high Zn exposure reduced its expression. Heterologous expression in yeast showed that SaPCR2 was responsible for Zn influx into the cells. The overexpression of *SaPCR2* in NHE *S. alfredii* increased root uptake and the root-to-shoot translocation of Zn, but did not influence the Mn, Cu or Fe concentrations. Furthermore, SR-μ-XRF analysis showed a similar tendency, and more Zn was distributed in the vascular buddle tissues as well as in the cortex and epidermis in the transgenic lines. Root morphology was also altered after *SaPCR2* overexpression, and a more severe inhibition was observed in the transgenic lines. In transgenic lines, the meristematic and elongation zones of the root were lower compared to the WT type, and Zn accumulation in the meristem cells was also reduced. Overall, these results suggest that SaPCR2 is an important transporter responsible for Zn uptake in roots and mainly functions in the root elongation zone of the *S. alfredii*. This research on *SaPCR2* could provide a theoretical basis for the use of genetic engineering technology, such as overexpressing SaPCR2 in crops to improve Zn accumulation while reducing Cd uptake for safe production and biological enhancement. In the future, transgenic plants including *SaPCR2* knockout lines and *SaPCR2* overexpression lines of HE *S. alfredii* need to be constructed to further clarify the role of SaPCR2 in metal transport.

## Data Availability

Not applicable.

## Supplementary Materials

The following supporting information can be downloaded at: https://www.mdpi.com/article/10.3390/life12050768/s1, Figure S1: Trace elements uptake including Zn, Mn, Cu, and Fe in *SaPCR2* transgenic lines; Figure S2: Root tip structures of *SaPCR2* transgenic lines grown in normal nutrient solution; Figure S3: Alignment of protein sequences of SaPCR2, AtZIP1, AtZIP2, and NcZNT1. Red box indicated a highly conserved sequence region; Table S1: Primers for real-time PCR analysis and vector construction of *SaPCR2*.
