# Supplementary material for: Role of SaPCR2 in Zn Uptake in the Root Elongation Zone of the Zn/Cd Hyperaccumulator Sedum alfredii
Source: Life (Basel). 2022 May 23;12(5):768. doi: 10.3390/life12050768 (PMC9146221; doi:10.3390/life12050768)
Supplement: Supplementary file 1 [file life-12-00768-s001.zip › life-1705224-supplementary.pdf]

life

MDPI

Table S1. Primers for real-time PCR analysis and vector construction of *SaPCR2*.

Primer ID	Primer sequence (5′ to 3′)	Function	Reference
SaACTIN1-qF1	TGTGCTTTCCCTCTATGCC	Real-time PCR	[1]
SaACTIN1-qR1	CTGCTCCATACAAGCCAACCACG		
SaPCR2-qF1	GCGGTGGGATGTGGTCTAC	Real-time PCR	[2]
SaPCR2-qR1	CGATAATCTCGGCTATTTGGC		
pDR196-SaPCR2-F1	tataccccagcctcgactagtATGTATCCATCTTTGTCTG	Vector construction	[2]
pDR196-SaPCR2-R1	gataagcttgatatcgaattcTCATCTACTCATCCCCTGC		

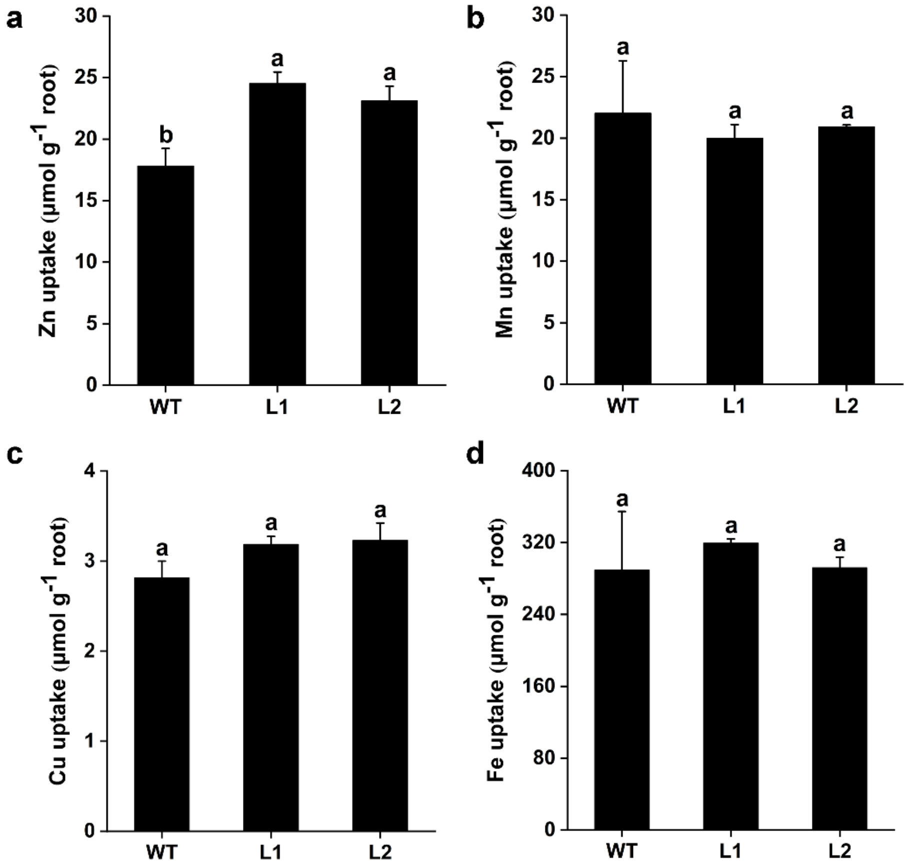

Figure S1. Trace elements uptake, including (**a**) Zn, (**b**) Mn, (**c**) Cu, and (**d**) Fe, in the non-hyperaccumulating ecotype (NHE) *Sedum alfredii* wild type (WT) and *SaPCR2* overexpressing lines (L1 and L2) after five-week-preculturing. Uptake was calculated in terms of the root dry weight. Different letters indicate significant differences between genotype in a treatment ($P < 0.05$). Error bars = mean ± standard error (SE; n = 3).

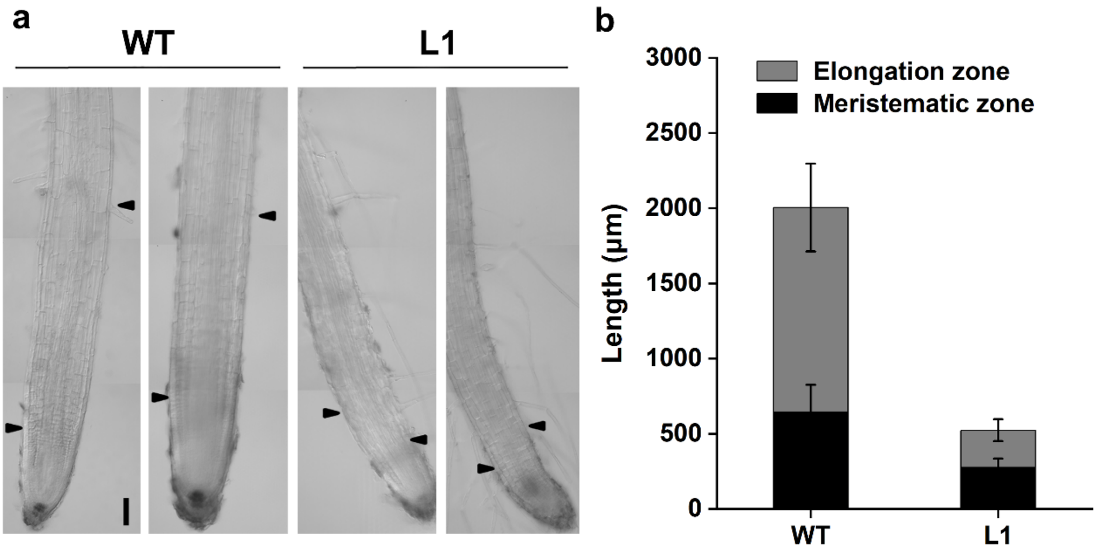

Figure S2. Root tip structures of the non-hyperaccumulating ecotype (NHE) *Sedum alfredii* wild type (WT) and *SaPCR2* overexpressing lines (L1 and L2) grown in normal nutrient solution. (**a**) Growth of root tips. Bar=200 μm. (**b**) Length of meristematic zone and elongation zone. Error bars = mean ± SE (n=5).

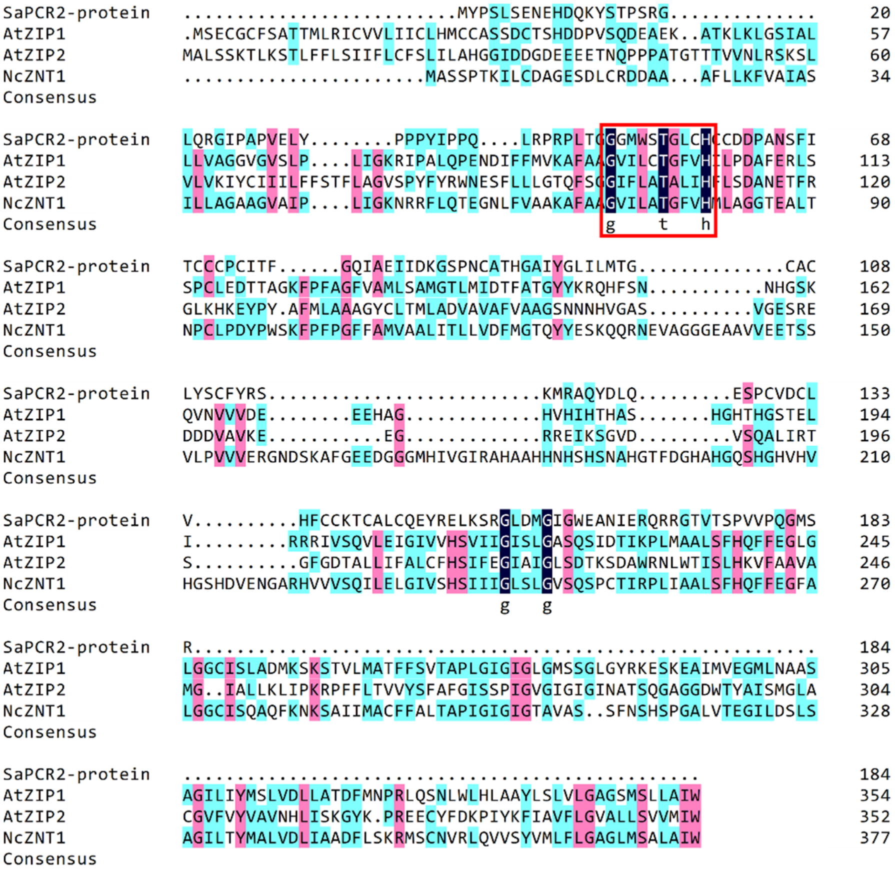

Figure S3. Alignment of protein sequences of SaPCR2, AtZIP1, AtZIP2, and NcZNT1. Red box indicated a highly conserved sequence region.

References

1. Chao, Y.; Zhang, M.; Feng, Y.; Yang, X.; Islam, E. cDNA-AFLP analysis of inducible gene expression in zinc hyperaccumulator *Sedum alfredii* Hance under zinc induction. *Environ. Exp. Bot.* **2010**, *68*, 107-112, https://doi:10.1016/j.envexpbot.2009.11.013.
2. Lin, J.; Gao, X.; Zhao, J.; Zhang, J.; Chen, S.; Lu, L. Plant Cadmium Resistance 2 (SaPCR2) facilitates cadmium efflux in the roots of hyperaccumulator *Sedum alfredii* Hance. *Front. Plant Sci.* **2020**, *11*, 568887, https://doi:10.3389/fpls.2020.568887.
